# Arf6 exacerbates allergic asthma through cell-to-cell transmission of ASC inflammasomes

**DOI:** 10.1172/jci.insight.139190

**Published:** 2021-08-23

**Authors:** SangJoon Lee, Akari Ishitsuka, Takahiro Kuroki, Yu-Hsien Lin, Akira Shibuya, Tsunaki Hongu, Yuji Funakoshi, Yasunori Kanaho, Kyosuke Nagata, Atsushi Kawaguchi

**Affiliations:** 1 Department of Infection Biology, Faculty of Medicine,; 2 PhD Program in Human Biology, School of Integrative and Global Majors,; 3 Graduate School of Comprehensive Human Sciences,; 4 Department of Immunology, Faculty of Medicine,; 5 Department of Physiological Chemistry, Faculty of Medicine,; 6 Transborder Medical Research Center, and; 7 Microbiology Research Center for Sustainability, University of Tsukuba, Tsukuba, Japan.

**Keywords:** Inflammation, Asthma

## Abstract

Asthma is a chronic inflammatory disease of the airways associated with excess production of Th2 cytokines and lung eosinophil accumulation. This inflammatory response persists in spite of steroid administration that blocks autocrine/paracrine loops of inflammatory cytokines, and the detailed mechanisms underlying asthma exacerbation remain unclear. Here, we show that asthma exacerbation is triggered by airway macrophages through a prion-like cell-to-cell transmission of extracellular particulates, including ASC protein, that assemble inflammasomes and mediate IL-1β production. OVA-induced allergic asthma and associated IL-1β production were alleviated in mice with small GTPase *Arf6*-deficient macrophages. The extracellular ASC specks were slightly engulfed by *Arf6*^–/–^ macrophages, and the IL-1β production was reduced in *Arf6*^–/–^ macrophages compared with that in WT macrophages. Furthermore, pharmacological inhibition of the Arf6 guanine nucleotide exchange factor suppressed asthma-like allergic inflammation in OVA-challenged WT mice. Collectively, the Arf6-dependent intercellular transmission of extracellular ASC specks contributes to the amplification of allergic inflammation and subsequent asthma exacerbation.

## Graphical abstract

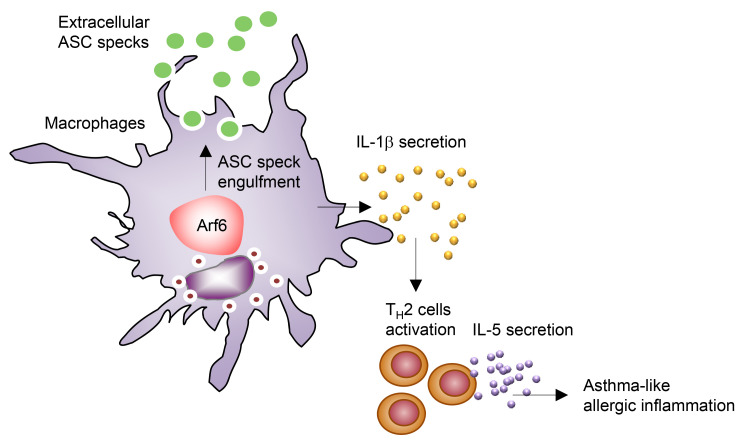

## Introduction

Asthma causes approximately 250,000 deaths annually, and 300 million people suffer from the disease worldwide (1). Airway hyperresponsiveness and airway inflammation are the major symptoms of the disease and are generally exacerbated by Th 2 cells through production of type 2 cytokines, including IL-5 and IL-13. These type 2 cytokines are responsible for the production of allergen-specific IgE, eosinophil migration, and airway abnormalities, such as mucous cell metaplasia (2, 3).

Airway macrophages provide the first line of host defense against a variety of external stimuli, including antigens, pollutions, and infectious agents, in the respiratory mucosal surface. Airway macrophages are crucial for maintaining tissue homeostasis by the induction of innate immune responses and clearance of both particulate substances and dying cells from the airways through their high phagocytic activity. In spite of this, the role of airway macrophages in the exacerbation of allergic asthma remains controversial. It is reported that airway macrophages suppress inflammatory responses and neutralize the Th2 response through enhanced production of IL-10 and IL-12 in asthma (4). However, airway macrophages have been implicated in the development and progression of allergic asthma through the production of proinflammatory cytokines, including IL-1β, TNF-α, IL-8, and IL-17 (5).

Emerging evidence shows excess IL-1β release in patients with severe asthma (6), suggesting that IL-1β pathway inhibition is an attractive therapeutic target for severe asthma. Exposure to typical triggers, such as allergens and irritant chemicals, induces IL-1β production from macrophages that stimulate the differentiation of Th2 cells (7–12). IL-1β production in macrophages is controlled through proteolytic maturation of pro–IL-1β mediated by inflammasomes, which are multiprotein complexes consisting of caspase-1, apoptosis-associated speck-like protein containing a CARD (ASC), and NOD-like receptor family proteins, such as NLRP3 (13). NLRP3 is an intracellular sensor protein expressed in macrophages and activated by a wide variety of microbes. NLRP3 also recognizes large particulate matters as damage-associated molecular patterns (DAMPs), including aluminium potassium (alum), silica, uric acid, and amyloid β, after engulfment in macrophages. The activated NLRP3 then facilitates ASC oligomerization to form large, intracellular macromolecular aggregates called ASC specks. Although the exact molecular mechanism is unclear, *Nlrp3*^–/–^ mice were observed to have decreased pulmonary inflammation, suppressed mucus secretion, and decreased levels of Th2 response and IgE production in a mouse model of OVA-induced allergic asthma (14). It is reported that ASC specks are released into the extracellular space and the extracellular ASC can propagate inflammatory responses through a prion-like transmission mediated by engulfment in macrophages (15, 16). Notably, increased extracellular ASC specks are observed in the bronchoalveolar lavage fluid (BALF) of patients with chronic obstructive pulmonary disease and pneumonia (15, 16).

The small GTPase ADP-ribosylation factor (Arf) family members are key regulators of various types of intracellular trafficking (17). Arf6 is unique among other Arf proteins in its ability to stimulate the rearrangement of cortical actin to regulate plasma membrane dynamics, including phagocytosis (18, 19). Arf6 activates phosphatidylinositol-4-phosphate 5-kinase (PIP5K) to generate PI(4,5)P2, and then PI(4,5)P2 regulates the cortical actin polymerization (18). Here, we found that OVA-induced allergic asthma was alleviated in mice with macrophages rendered phagocytosis deficient by conditional knockout (cKO) of *Arf6* gene. The extracellular ASC specks were hardly transmitted to *Arf6*^–/–^ airway macrophages, resulting in less IL-1β production. Furthermore, pharmacological inhibition of Arf6 activation by SecinH3, a potent inhibitor of Arf6 guanine nucleotide exchange factor (GEF), suppresses bronchiolitis with mucus hypersecretion induced by OVA challenge. Collectively, we propose that Arf6 is required for the engulfment of extracellular ASC specks in airway macrophages and contributes to the exacerbation of asthma-like allergic inflammation.

## Results

### Asthma exacerbation is alleviated in macrophage-Arf6 cKO mice.

**Figure 1:**
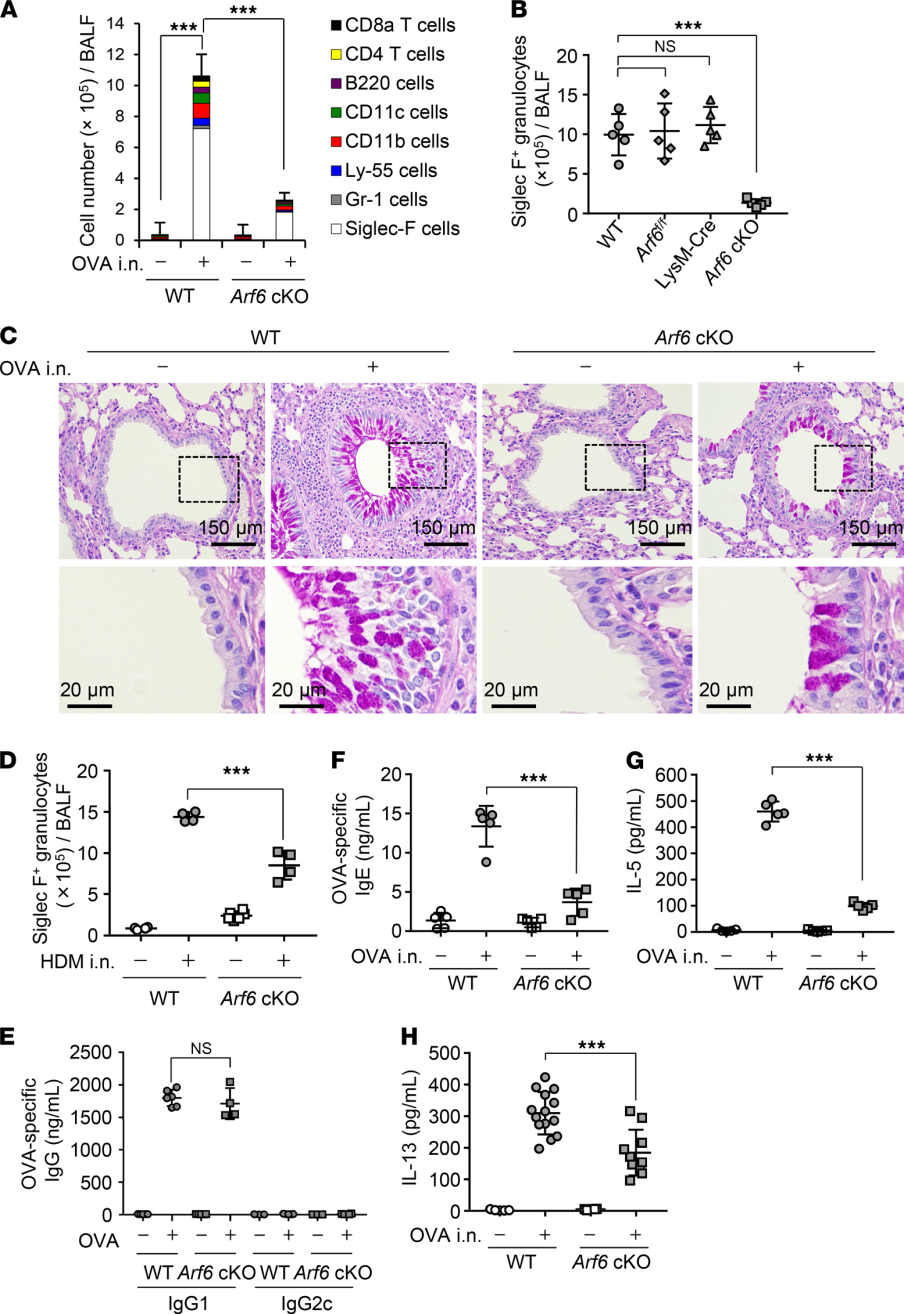
Asthma exacerbation is alleviated in macrophage-*Arf6* cKO mice. WT and macrophage-*Arf6* cKO mice were immunized by intraperitoneal injection of OVA with aluminium hydroxide as an adjuvant once per week for 3 weeks. At days 7, 10, and 13 after the last immunization, the mice received intranasal injection of OVA. (**A**) At day 1 after the last OVA challenge, the number of each indicated leukocyte subset in BALF obtained from WT and macrophage-*Arf6* cKO (*Arf6* cKO) mice was examined by FACS (*n* = 3 mice per group; mean ± SD are shown). ***P < 0.001, 1-way ANOVA with Tukey’s test for CD45.2-positive cells. (**B**) The number of Siglec-F–positive granulocytes in BALF obtained from WT and macrophage-*Arf6* cKO mice was examined by FACS (*n* = 5 mice per group). The combined results from 2 independent experiments are shown. ***P < 0.001; 1-way ANOVA with Tukey’s test. (**C**) Lung tissue sections were stained with PAS-hematoxylin at day 1 after the last OVA challenge. Data are representative of 3 independent experiments. Scale bar: 150 μm (top); 20 μm (bottom). (**D**) The number of Siglec-F–positive granulocytes in BALF obtained from WT and macrophage-*Arf6* cKO mice challenged with HDM was examined by FACS (*n* = 5 mice per group). ***P < 0.001, 2-tailed Student’s *t* test. (**E–H**) The amount of OVA-specific IgG1 in serum (**E**), OVA-specific IgE in BALF (**F**), IL-5 in BALF (**G**), and IL-13 in BALF (**H**) obtained from the indicated mice was examined by ELISA (*n* = 5–14 mice per group). Each symbol represents 1 mouse. The combined results from 2 independent experiments are shown. ***P < 0.001, 2-tailed Student’s *t* test.

To elucidate the molecular mechanism of asthma exacerbation mediated by airway macrophages, we generated macrophage-selective *Arf6*-deficient mice (macrophage-*Arf6* cKO) by mating *Arf6^fl/fl^* mice with LysM-Cre mice. Arf6 regulates the membrane dynamics important for the phagocytosis through the reorganization of the actin cytoskeleton. WT and macrophage-*Arf6* cKO mice were sensitized by intraperitoneal injection of OVA with aluminium hydroxide on days 1, 7, and 14. Seven days after the last immunization, allergic asthma-like responses were induced by intranasal administration of OVA. We then examined the number of infiltrating leukocytes in BALF by FACS analysis with anti-CD8a, anti-CD4, anti-B220, anti-CD11c, anti-CD11b, anti-NK1.1, anti–Gr-1, and anti–Siglec F antibodies. Although the various leukocyte subsets had migrated in the lungs of WT mice, the number of Siglec F–positive cells was most increased by the intranasal administration of OVA, indicating that eosinophil-mediated allergic inflammation was induced (Figure 1A). However, the number of Siglec F–positive granulocytes in each infiltrating leukocyte subset was substantially reduced in macrophage-*Arf6* cKO mice compared with that in WT, *Arf6^fl/fl^*, and LysM-Cre mice (Figure 1B). Histological analysis of lung slices of WT mice showed a massive infiltration of eosinophils and mononuclear inflammatory cells concomitant with mucus hypersecretion and thickening of the peribronchial epithelium (Figure 1C). In contrast, macrophage-*Arf6* cKO mice showed no severe pathogenic changes upon OVA challenge (Figure 1C). Similar results were obtained for the eosinophil infiltration and the histological analysis in house dust mite–induced (HDM-induced) asthma mouse model (Figure 1D and Supplemental Figure 1; supplemental material available online with this article; https://doi.org/10.1172/jci.insight.139190DS1).

IgG1 and IgG2c are indicators for preferential Th2 and Th1 responses, respectively. The level of OVA-specific IgG1 antibody in sera obtained from macrophage-*Arf6* cKO mice was comparable to that from WT mice, suggesting that the cKO of Arf6 does not impair OVA sensitization before OVA challenge (Figure 1E). Furthermore, the expression of OVA-specific IgG2c was not induced by OVA sensitization in WT and macrophage-*Arf6* cKO mice (Figure 1E). This indicates that the Th2 response was successfully induced by the OVA sensitization in macrophage-*Arf6* cKO mice as well as WT mice. We also performed the intranasal administration of Alexa Fluor 488–conjugated OVA (Supplemental Figure 2). We found that the number of Alexa Fluor 488–positive dendritic cells in mediastinal lymph nodes was comparable between WT and macrophage-*Arf6* cKO mice, suggesting that *Arf6* deletion by LysM Cre did not affect the antigen presentation by dendritic cells (Supplemental Figure 2). However, the amount of OVA-specific IgE (Figure 1F), IL-5 (Figure 1G), and IL-13 (Figure 1H) in BALF obtained from macrophage-*Arf6* cKO mice decreased compared with that obtained from WT mice, suggesting that macrophages exacerbate asthma-like allergic inflammation through Arf6. Although the production of both IgG1 and IgE is regulated by the Th2 response, the production of IgE in BALF (Figure 1F), but not IgG1 in sera (Figure 1E), was reduced in macrophage-*Arf6* cKO mice. Mice were sensitized by intraperitoneal injection of OVA with alum as an adjuvant, but the alum was not intranasally injected in OVA challenge. Alum adjuvant artificially stimulates a local inflammatory response by NLRP3 for immunopotentiation. Thus, it is possible that OVA administration alone is not enough for the Th2 response in macrophage-*Arf6* cKO mice, possibly due to a weak inflammatory response compared with that WT mice. Notably, the number of IL-13–positive ILC2 cells in macrophage-*Arf6* cKO mice was weakly increased by intranasal administration of OVA, as in WT mice (Supplemental Figure 3). This may cause the low level of goblet cell hyperplasia observed in the bronchi of macrophage-*Arf6* cKO mice (Figure 1C).

**Figure 2:**
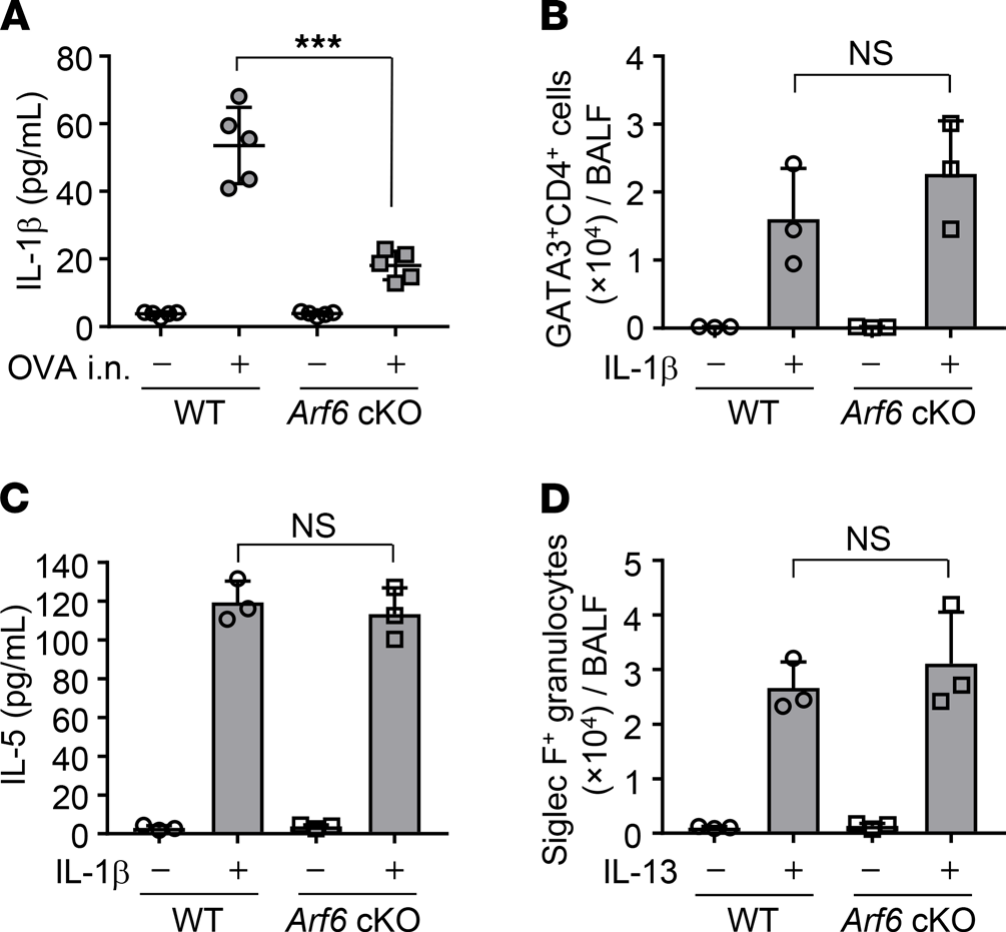
Arf6 is required for IL-1β production from airway macrophages in asthma-like allergic inflammation. (**A**) OVA-immunized WT and macrophage-*Arf6* cKO mice were intranasally challenged with OVA. The amount of IL-1β in BALF of indicated mice was examined by ELISA (*n* = 5 mice per group). Each symbol represents 1 mouse. The combined results from 2 independent experiments are shown. ***P < 0.001, 2-tailed Student’s *t* test. (**B** and **C**) WT and macrophage-*Arf6* cKO mice were intranasally injected with 1 μg recombinant murine IL-1β. At day 3 after injection, the number of GATA3^+^CD4^+^ leukocytes (**B**) and the amount of IL-5 (**C**) in BALF was examined by FACS and ELISA, respectively (*n* = 3 mice per group; mean ± SD are shown). (**D**) WT and macrophage-*Arf6* cKO mice were intranasally injected with 1 μg recombinant murine IL-13. At day 3 after injection, the number of Siglec-F–positive granulocytes in BALF was examined by FACS (*n* = 3 mice per group; mean ± SD are shown).

*Arf6 is required for IL-1βproduction from airway macrophages in asthma-like allergic inflammation*. Airway macrophages produce several proinflammatory cytokines upon allergen exposure (5). Among the proinflammatory cytokines, NLRP3 inflammasome-mediated IL-1β secretion induces allergic inflammation and activates OVA-specific T cell expansion and IgE production (20, 21). Given the Arf6-dependent asthma-like allergic inflammation, we next examined IL-1β secretion in the OVA-challenged macrophage-*Arf6* cKO mice. The amount of IL-1β in BALF obtained from macrophage-*Arf6* cKO mice decreased to about 30% of WT mouse levels (Figure 2A).

Arf6 regulates not only phagocytosis but also signal transduction and cell migration by reorganizing the actin cytoskeleton (22–24). We next examined the number of Th2 cells in BALF from macrophage-*Arf6* cKO mice intranasally treated with recombinant IL-1β (Figure 2B). FACS analysis with anti-GATA3 and anti-CD4 antibodies revealed that the number of infiltrating Th2 cells activated by IL-1β administration in macrophage-*Arf6* cKO mice was not different from that of WT mice (Figure 2B). This suggests that Th2 activation ability is not impaired in mice lacking the *Arf6* gene through the expression of Cre recombinase driven by *LysM* promoter. Furthermore, the production of the Th2 cytokine IL-5 from macrophage-*Arf6* cKO mice treated with IL-1β was comparable to that of WT mice (Figure 2C). These findings indicate that the proinflammatory function of Th2 cells stimulated by IL-1β is still intact even in macrophage-*Arf6* cKO mice. Note that the migration of eosinophils in lungs stimulated by intranasal injection of IL-13 (25–27) was unchanged in macrophage-*Arf6* cKO mice compared with WT mice (Figure 2D).

**Figure 3:**
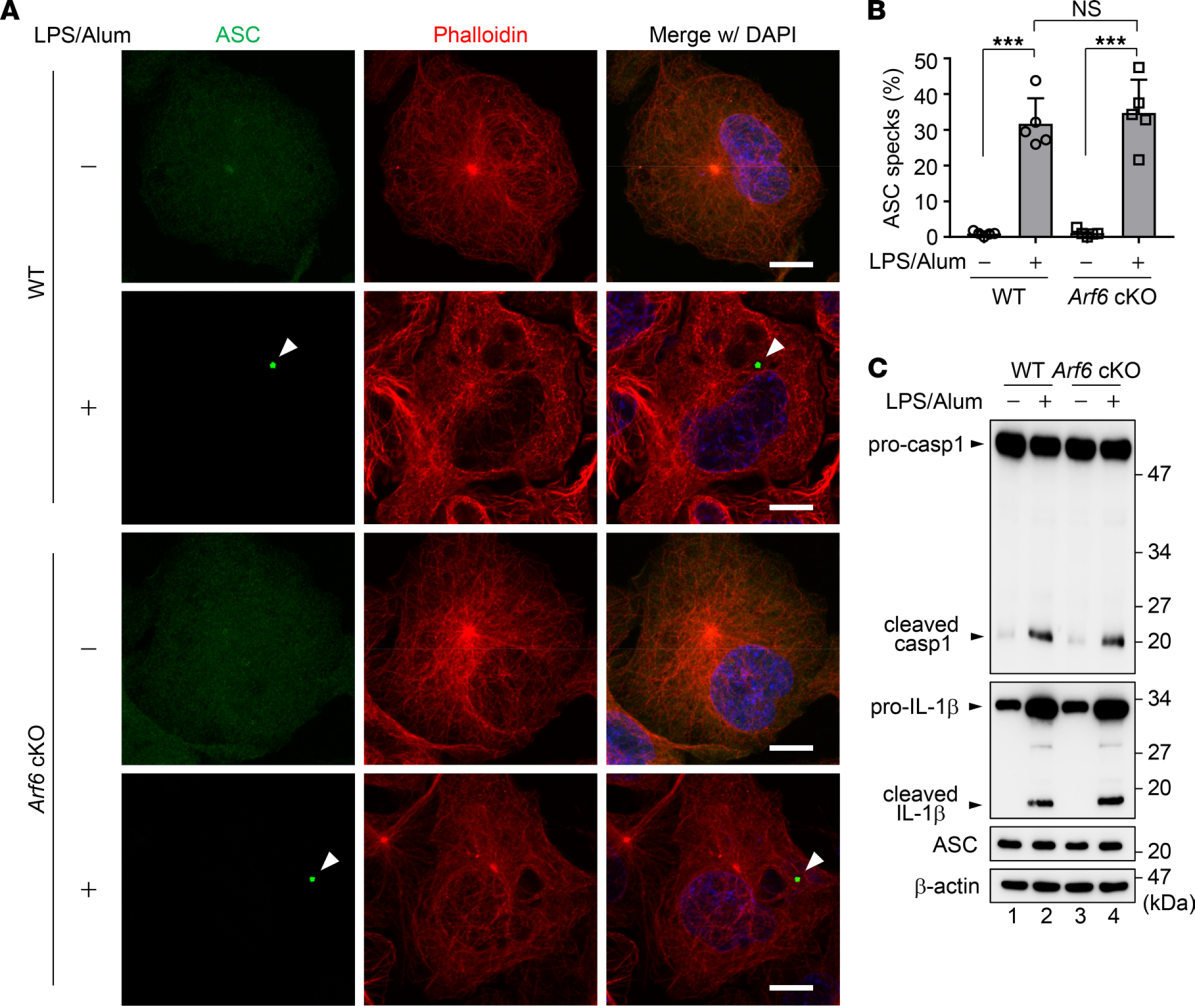
The primary inflammasome activation is not impaired in *Arf6*^–/–^ macrophages. (**A** and **B**) Airway macrophages were isolated from WT and macrophage-*Arf6* cKO mice and then subjected to indirect immunofluorescence assays with anti-ASC (green) antibody and Alexa Fluor 568 phalloidin (red) at 36 hours after treatment with 200 ng/ml LPS and 250 μg/ml alum (**A**). Arrowheads indicate ASC specks. Scale bar: 10 μm. Data are representative of 3 independent experiments. The number of cells showing ASC specks was counted (**B**) (*n* > 100; mean ± SD from 5 independent experiments). ***P < 0.001, 1-way ANOVA with Tukey’s test. (**C**) At 36 hours after treatment with 200 ng/ml LPS and 250 μg/ml alum, the expression levels of caspase-1 and IL-1β in WT and *Arf6*^–/–^ macrophages were examined. Data are representative of 3 independent experiments.

*Arf6 is required for the engulfment of extracellular ASC specks to activate the allergen-independent IL-1βsecretion*. Upon ligand recognition, NLRP3 forms a multiprotein complex with the adaptor protein ASC and caspase-1 to form inflammasome complexes, which leads to the proteolytic activation of IL-1β (13). To elucidate how Arf6 stimulates IL-1β production, the formation of inflammasomes was examined in vitro in airway macrophages isolated from WT and macrophage-*Arf6* cKO mice. The expression of *Arf6* was not observed in airway macrophages obtained from macrophage-*Arf6* cKO mice (Supplemental Figure 4, A and B). We found that intracellular ASC specks were formed in about 30% of *Arf6*^–/–^ macrophages by LPS and alum treatment, in line with the WT macrophages (Figure 3, A and B). The knockout of *Arf6* also did not impair either the expression of pro–IL-1β and procaspase-1 or their proteolytic activation (Figure 3C). These findings suggest that the primary activation of inflammasomes does not require Arf6 in macrophages.

**Figure 4:**
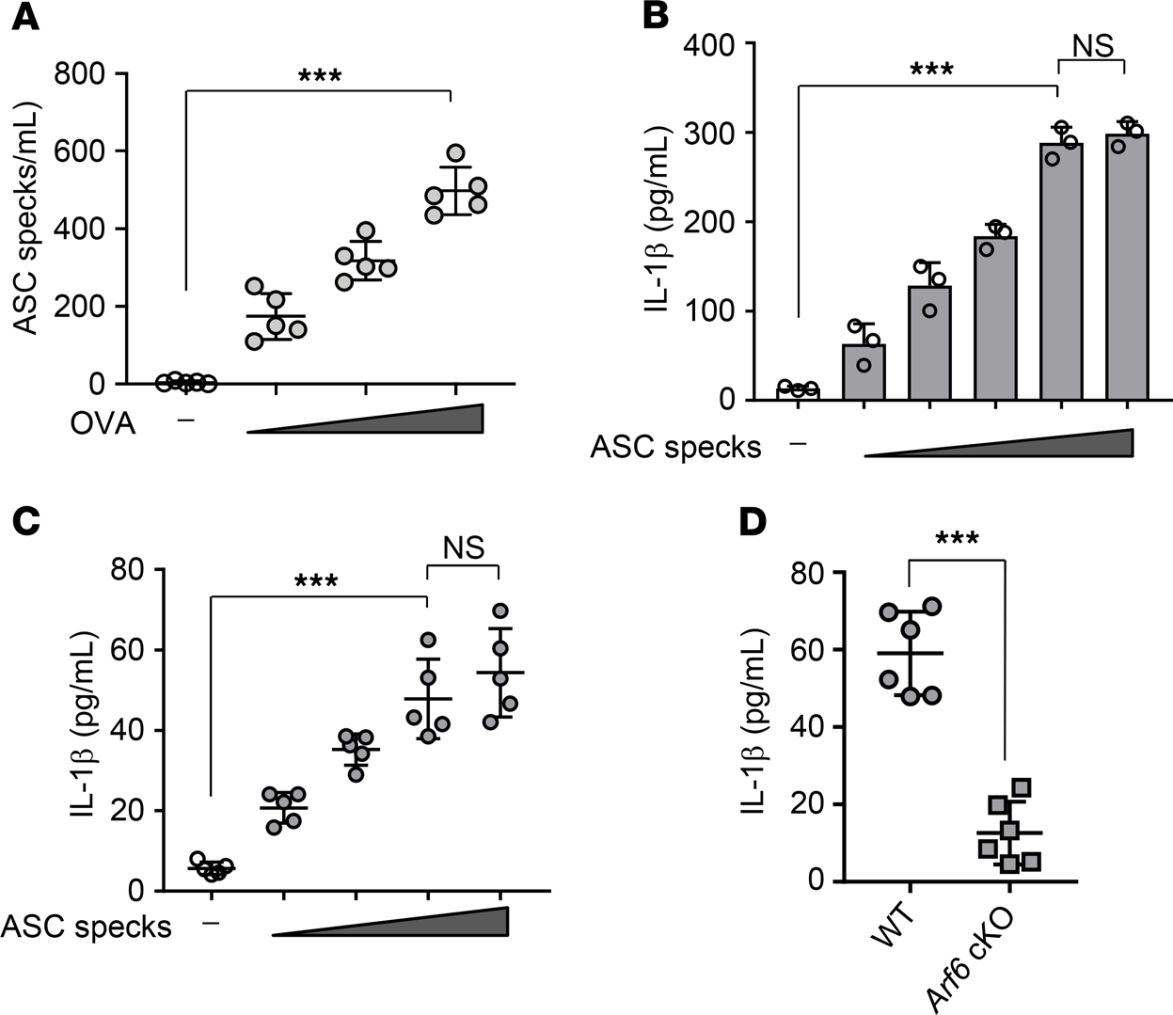
Arf6 is required for extracellular ASC speck–mediated IL-1β secretion. (**A**) After OVA challenge, BALF was obtained from WT mice and subjected to Percoll centrifugation to isolate extracellular ASC specks. The number of ASC specks was counted (*n* = 5 mice per group; the combined results from 2 independent experiments are shown). Each symbol represents 1 mouse. ***P < 0.001, 1-way ANOVA with Tukey’s test. (**B**) IL-1β secretion in airway macrophages isolated from WT mice was examined by ELISA at 6 hours after treatment with 5 × 10^2^, 1 × 10^3^, 5 × 10^3^, 1 × 10^4^, or 5 × 10^4^ particles of purified GFP-ASC specks. ***P < 0.001, 1-way ANOVA with Tukey’s test (mean ± SD from 3 independent experiments). (**C**) WT mice received intranasal injection of 5 × 10^5^, 1 × 10^6^, 5 × 10^6^, or 1 × 10^7^ particles of purified GFP-ASC specks. The amount of IL-1β in BALF of WT mice was examined by ELISA at day 3 after injection (*n* = 5 mice per group; the combined results from 2 independent experiments are shown). Each symbol represents 1 mouse. ***P < 0.001, 1-way ANOVA with Tukey’s test. (**D**) WT and macrophage-*Arf6* cKO mice were intranasally injected with 5 × 10^6^ particles of purified GFP-ASC specks. The amount of IL-1β in BALF obtained from the indicated mice was examined by ELISA at day 3 after injection (*n* = 6 mice per group; the combined results from 2 independent experiments are shown). Each symbol represents 1 mouse. ***P < 0.001, 2-tailed Student’s *t* test.

Emerging evidence demonstrates that ASC specks are released into the extracellular space where they are then engulfed via neighboring macrophages to activate ASC oligomerization by a prion-like mechanism in the absence of any allergens or infectious agents (15, 16). We also found that the intranasal administration of OVA increased the number of extracellular ASC specks in BALF obtained from WT mice in a dose-dependent manner (Figure 4A). To address the involvement of Arf6 in the extracellular ASC speck–mediated inflammatory response, we examined IL-1β production induced by adding extracellular ASC specks in vitro and in vivo. The extracellular ASC specks were purified by Percoll gradient centrifugation from THP-1–derived macrophages expressing GFP-fused ASC (GFP-ASC) after LPS and alum treatment (Supplemental Figure 5). These purified ASC specks could induce IL-1β secretion both in vitro in airway macrophages obtained from WT mice (Figure 4B) and in vivo in BALF from WT mice through the intranasal injection (Figure 4C). In contrast, the amount of IL-1β in BALF obtained from macrophage-*Arf6* cKO mice decreased to about 30% of that from WT mice at day 3 after intranasal injection of purified ASC specks (Figure 4D).

**Figure 5:**
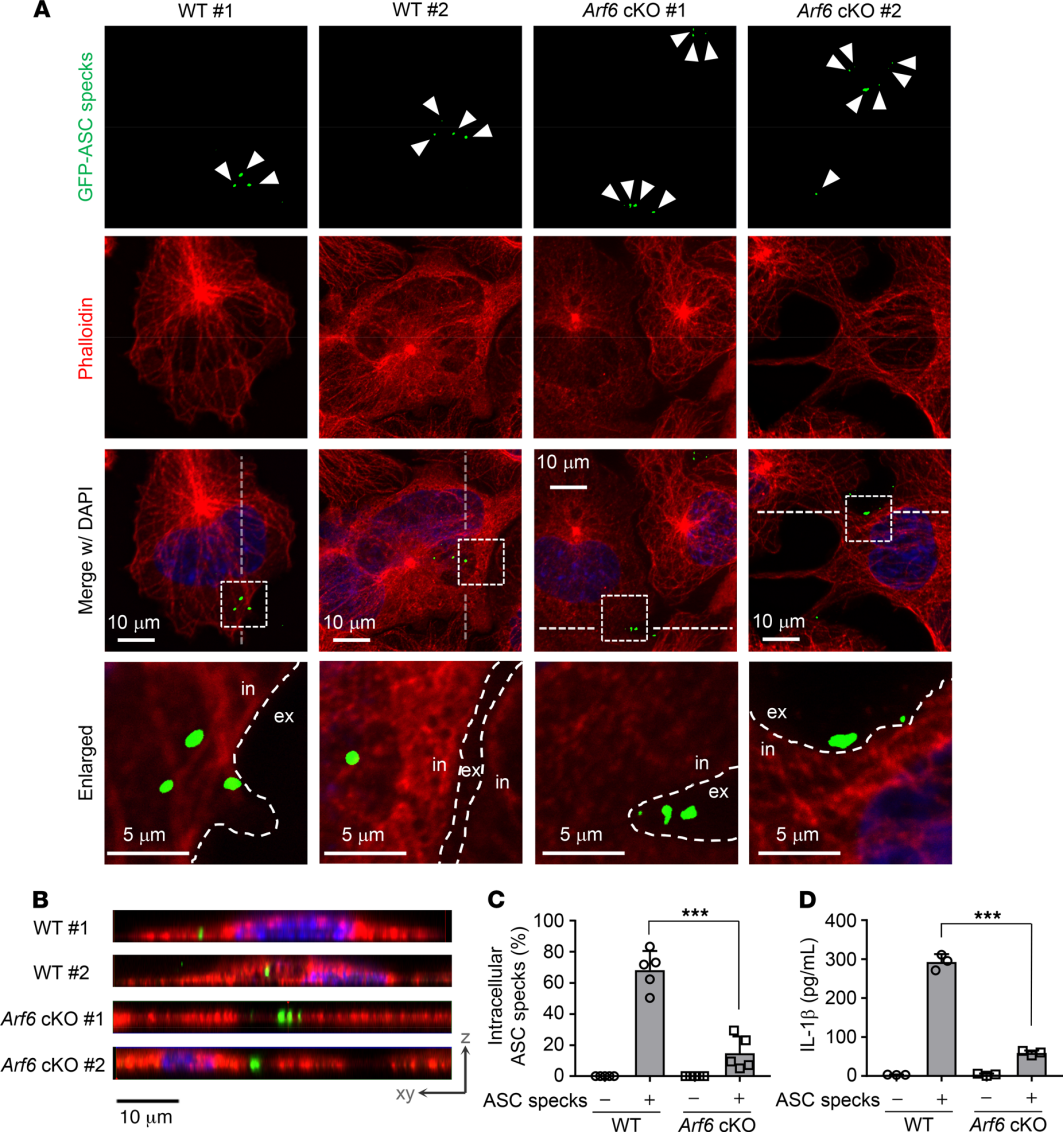
Engulfment of extracellular ASC specks is mediated by Arf6 for allergen-independent IL-1β production. (**A–C**) WT and *Arf6*^–/–^ macrophages were stained with Alexa Fluor 568 phalloidin (red) at 6 hours after treatment with 1 × 10^4^ particles of purified GFP-ASC specks (scale bar: 10 μm [top 3 rows]; 5 μm [bottom]) (**A**). Arrowheads indicate ASC specks. in, intracellular; ex, extracellular. Data are representative of 3 independent experiments. Two different fields are shown in each sample. The vertical section images from *Z*-stack series were reconstructed. Scale bar: 10 μm. (**B**). Dotted lines indicate the position of the reconstituted vertical plane. The number of cells showing intracellular ASC specks was counted (**C**) (*n* > 100; mean ± SD from 5 independent experiments). ***P < 0.001, 2-tailed Student’s *t* test. (**D**) At 6 hours after treatment of purified GFP-ASC specks, the amount of IL-1β secretion in WT or *Arf6*^–/–^ macrophages was examined by ELISA. ***P < 0.001, 2-tailed Student’s *t* test. Mean ± SD from 3 independent experiments.

Next, to examine the phagocytosis of ASC specks by macrophages, WT or *Arf6*^–/–^ airway macrophages were incubated with purified extracellular GFP-ASC specks and stained with Alexa Fluor 568 phalloidin to mark the cytoplasm. We found that about 65% of WT airway macrophages engulfed GFP-ASC specks (Figure 5, A–C) and the engulfment was concomitant with substantial IL-1β secretion (Figure 5D). In contrast, only about 15% of *Arf6*^–/–^ airway macrophages engulfed GFP-ASC specks (Figure 5, A–C), and they secreted a smaller amount of IL-1β compared with WT airway macrophages (Figure 5D). These findings suggest that Arf6 is required for the engulfment of extracellular ASC specks by macrophages for IL-1β secretion without any allergen treatment. It has been reported that LysM Cre is expressed in macrophages and neutrophils (28). However, in our mouse line, Arf6 was expressed in neutrophils isolated from bone marrow of macrophage-*Arf6* cKO mice (Supplemental Figure 6A), and IL-1β production induced by adding ASC specks was comparable between WT and macrophage-*Arf6* cKO mice (Supplemental Figure 6B).

### SecinH3 suppresses asthma-like allergic inflammation.

**Figure 6:**
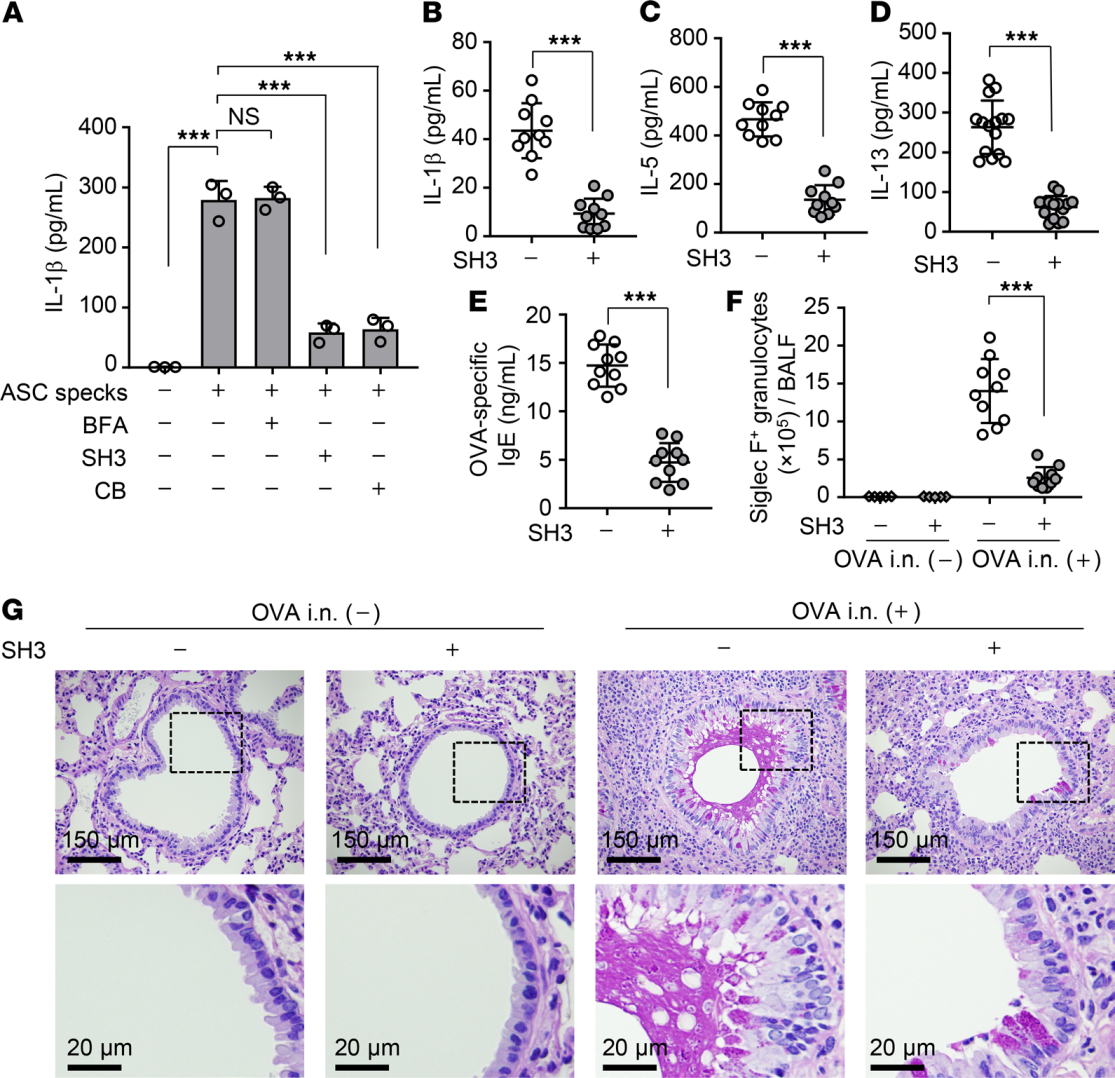
SecinH3 suppresses asthma-like allergic inflammation. (**A**) Airway macrophages obtained from WT mice were treated with 100 μM brefeldin A (BFA), 50 μM SecinH3 (SH3), or 30 μM cytochalasin B (CB). At 3 hours after incubation, cells were further incubated with 1 × 10^4^ particles of purified GFP-ASC specks. The level of secreted IL-1β was examined by ELISA at 6 hours after treatment of purified GFP-ASC specks. ***P < 0.001, 1-way ANOVA with Tukey’s test (mean ± SD from 3 independent experiments). (**B–G**) OVA-immunized WT mice were intranasally injected with OVA at day 7 after the last immunization. After a 1-day incubation, the mice were intranasally administered with 50 nmol/head SecinH3 and then challenged with OVA at days 10 and 13 after the last immunization. The amount of IL-1β (**B**), IL-5 (**C**), IL-13 (**D**), and OVA-specific IgE (**E**) and the number of Siglec-F–positive granulocytes (**F**) in BALF were examined at day 1 after the last OVA challenge by ELISA and FACS, respectively (*n* = 5–10 mice per group). Each symbol represents 1 mouse. ***P < 0.001, 2-tailed Student’s *t* test. The combined results from 2 independent experiments are shown. Lung tissue sections were stained with PAS-hematoxylin at day 1 after the last OVA challenge (**G**). Scale bar: 150 μm (top); 20 μm (bottom). Data are representative of 3 independent experiments.

The cytohesin family proteins function as GEF that activates Arf6 by exchanging GDP to GTP (19). Next, IL-1β secretion in WT airway macrophages treated with SecinH3, a potent Arf6 GEF inhibitor (29), was examined via extracellular ASC speck stimulation. SecinH3 treatment reduced the amount of IL-1β secreted from WT airway macrophages to about 20% of that without SecinH3 treatment (Figure 6A, lane 4). Similar results were obtained from WT airway macrophages treated with cytochalasin B, which impairs phagocytosis by inhibiting actin polymerization (Figure 6A, lane 5). Notably, brefeldin A, which is a GEF inhibitor of other Arf family proteins, including Arf1, Arf3, and Arf5 (30, 31), did not inhibit the IL-1β secretion (Figure 6A, lane 3), suggesting that the engulfment of ASC specks is independent of Arf1, Arf3, and Arf5 proteins.

From these results, SecinH3 appears to be of use as a potential inhibitor for therapeutic intervention in allergic asthma. To address this possibility, we examined whether the pharmacological inhibition of Arf6 activation suppresses asthma-like allergic inflammation in vivo. OVA-immunized WT mice were intranasally injected with OVA, and, after a 1-day incubation, the mice were intranasally administered SecinH3 (50 nmol/head). At days 3 and 6 after the first OVA challenge, the mice further received intranasal OVA injections. SecinH3 administration reduced the amount of IL-1β in BALF obtained from OVA-challenged WT mice to about 20% of that without SecinH3 (Figure 6B). The amount of IL-5 secretion (Figure 6C), IL-13 secretion (Figure 6D), OVA-specific IgE production (Figure 6E), and infiltrating eosinophils (Figure 6F) in BALF was also impaired in response to the reduction of IL-1β through administration of SecinH3. As expected, bronchiolitis with mucus hypersecretion induced by OVA challenge was dramatically reduced by SecinH3 administration (Figure 6G). Furthermore, the effect of SecinH3 was dependent on the *Arf6* allele (Supplemental Figure 7, A and B). These findings indicate that the inhibition of Arf6 can be a therapeutic target against allergic asthma.

## Discussion

ASC consists of PYD and CARD domains. ASC oligomerizes into filaments through PYD/PYD interactions in a “prion-like” manner, and then, these filaments are condensed into macromolecular ASC specks through the CARD domain (32). ASC specks were observed extracellularly in patients with chronic respiratory diseases and autoinflammatory diseases, and the extracellular ASC specks are internalized by neighboring phagocytic cells, such as macrophages and dendritic cells, to induce inflammation (15, 16, 33), but its contribution to inflammation and diseases remains unclear. Here, we found that macrophages are responsible for the cell-to-cell propagation of extracellular ASC specks through Arf6-dependent phagocytosis, leading to the exacerbation of allergic asthma. In general, phagocytic cells recognize extracellular particles as a target through phagocytic receptors, such as C-type lectins and Fc receptors (34). Upon recognizing particles by phagocytic receptors, various signaling pathways are activated to initiate the reorganization of the actin cytoskeleton for phagocytosis in a phosphatidylinositol-4,5-bisphosphate [PI(4,5)P2]-dependent manner. PI(4,5)P2 is synthesized by PIP5K, which is activated by Arf6 (18), and regulates the activity of actin-binding proteins. Much less is known about the uptake mechanism of ASC specks, including whether it is mediated by phagocytic receptors. In addition, further studies are needed to reveal how ASC specks escape from phagolysosome to serve as a primer to polymerize ASC molecules in the cytoplasm of recipient cells.

Allergic asthma is triggered by exposure to allergens, which are generally captured by dendritic cells and activate Th2-mediated adaptive immunity. Although the exact roles of macrophages in the exacerbation of allergic asthma remain unclear, it is known that macrophages regulate both the proinflammatory response induced by a variety of foreign substances and the antiinflammatory response to avoid excessive tissue damage. Our study provides key evidence that asthma is exacerbated by IL-1β production from macrophages through a prion-like transmission of the extracellular ASC specks. Macrophages polarize into diverse phenotypes in response to signals derived from their environment. Thus, several classes of macrophages have been reported based on the expression of their cell surface markers, production of specific factors, and biological activities (35). However, as the phenotypic subsets of macrophages most involved in asthma exacerbation remain unclear, detailed characterization of the macrophage subsets that engulf extracellular ASC specks and accelerate allergic inflammation is needed.

Here, we demonstrated that Arf6 activation is required for the engulfment of extracellular ASC specks in airway macrophages, which leads to the exacerbation of allergic asthma. Among the Arf protein family, Arf6 is primarily involved in the actin dynamics (18). GEF proteins of the Arf family are classified into cytohesin, EFA6, BRAG, GBF1, and BIG subfamilies (36). Among these Arf GEFs, cytohesin family proteins, EFA6 family proteins, and BRAG2/GEP100 are responsible for the activation of Arf6 (36). SecinH3 is an Arf6 GEF inhibitor specific for the cytohesin family and BRAG2/GEP100 (29). Interestingly, systemic treatment of mice with SecinH3 suppresses the pulmonary metastases of glioma xenograft tumors (37) as well as neovascularization in melanoma and lung carcinoma tumors (38). We found that the nasal administration of SecinH3 reduced IL-1β production and ameliorated the exacerbation of allergic asthma (Figure 6). Although systemic treatment with SecinH3 induced hepatic insulin resistance in mice (29), inhaled SecinH3 treatment may be potentially useful for severe asthma cases, such as corticosteroid-resistant asthma. Furthermore, extracellular ASC specks are also causative agents of other inflammatory diseases, including chronic obstructive pulmonary disease (15), chronic infantile neurological cutaneous and articular syndrome (16), and Alzheimer’s disease (39). These inflammatory diseases could thus be expected to respond to pharmacological inhibition of Arf6 activation.

Corticosteroid treatment inhibits the expression of cytokines and chemokines, suppresses the recruitment and activation of inflammatory cells, and attenuates airway inflammation. Thus, corticosteroids are first-line drugs to disrupt the inflammatory loop for the treatment of acute asthma, regardless of atopic status or IgE allergen response. However, the asthmatic inflammatory response is persistent in spite of corticosteroid administration. Our findings will contribute to understanding the mechanisms of asthma exacerbation, as extracellular ASC specks can induce allergen-independent airway inflammation. It will also be interesting to analyze the relapse rates of allergic asthma in patients treated with corticosteroids and Arf6 inhibitors, including SecinH3.

## Methods

### Biological materials.

LPS (InvivoGen; tlrl-3pelps), alum (InvivoGen; tlrl-alk), recombinant murine IL-5 (PEPROTECH; 210-13), recombinant murine IL-1β (R&D Systems; 401-ML), SecinH3 (Abcam; ab145048), Brefeldin A (Wako; 022-15991), Cytochalasin B (Wako; 14930-96-2), OVA (MilliporeSigma; A2512-1G), HDM (*Dermatophagoides pteronyssinus*; LSL; LG-5449), and aluminium hydroxide gel (Wako; 012-24241) were purchased. Cytokines were measured by ELISA with mouse IL-1β (R&D Systems; MLB00C), mouse IL-5 (R&D Systems; M5000), mouse IL-13 (R&D Systems; M1300CB), mouse OVA-specific IgG1 antibody (Cayman Chemical; 500830), and mouse OVA-specific IgE antibody (BioLegend; 439807) kits according to the manufacturer’s protocols. Mouse monoclonal antibodies against β-actin (MilliporeSigma; A5441), caspase-1 (R&D Systems; MAB6215), IL-1β (Cell Signaling Technology; 3A6), and a rabbit polyclonal antibody against ASC (AdipoGen; AG-25B-0006) were purchased. Primary airway macrophages were isolated from BALF and were grown in RPMI 1640 with 10% FBS, 65 μg/ml penicillin, 100 μg/ml streptomycin, and 0.25 μg/ml fungizone.

### Mice.

LysM-Cre mice were gift from Satoru Takahashi (University of Tsukuba). The cKO mice in which the *Arf6* gene was deleted from macrophages (macrophage-*Arf6* cKO mic) were generated by mating *Arf6^fl/fl^* (38) mice with LysM-Cre mice.

### Immunization and intranasal administration of OVA.

The 8- to 12-week-old mice were sensitized by intraperitoneal injection of 100 μg/head OVA or 10 μg/head HDM emulsified in 1 mg aluminium hydroxide in a total volume of 100 μl on days 1, 7, and 14. At days 7, 10, and 13 after the last immunization, the mice were anesthetized by intraperitoneal injection of pentobarbital sodium and then were injected intranasally with 100 μg OVA or 50 μg HDM in 50 μl PBS. For the histology, four-μm-thick paraffin lung tissue sections were stained with Periodic acid–Schiff–hematoxylin (PAS-hematoxylin), and the samples were observed using BZ-X700 microscopy (KEYENCE).

### Flow cytometry.

Cells were collected from BALF and resuspended in a buffer containing 154 mM NH_4_Cl, 10 mM KHCO_3_, and 0.1 mM EDTA for red blood cell lysis for 5 minutes. After washing with PBS containing 2% FBS, cells were stained with anti-CD45.2 (Biolegend; 109808), anti–Siglec F (Invitrogen; 12-1702-82), anti–Gr-1 (BioLegend; 108412), anti-CD4 (Biolegend; 100516), anti-CD8a (Biolegend; 100708), anti-Mac-1 (CD11b) (Biolegend; 101208), anti-CD11c (Biolegend; 117307), anti-NK-1.1 (Ly-55) (Biolegend; 108707), anti-B220 (Biolegend; 103211), or anti-IgG2a (Biolegend; 407109) antibodies for 30 minutes on ice. To confirm the Th2 cell differentiation, cells were collected from BALF and fixed with 1% paraformaldehyde (PFA) and permeabilized with PBS containing 0.1% Triton X-100 and 0.2% bovine serum albumin for 3 minutes. The cells were then incubated with PE-conjugated anti-GATA3 (Invitrogen; 12-9966-42) and APC-conjugated anti-CD4 (Biolegend; 100516) antibodies for 1 hour. Flow cytometry analysis was performed using a Guava easyCyto flow cytometer (Merck Millipore).

### Purification of extracellular ASC specks.

THP-1 cells (Riken Cell Bank, Tsukuba, Japan) constitutively expressing GFP-ASC were prepared as previously described (40). THP-1 cells constitutively expressing GFP-ASC were grown in RPMI 1640 with 10% FBS and differentiated into macrophages in RPMI 1640 medium containing 20% FBS and 100 ng/ml PMA for 2 days. At 48 hours after treatment with 200 ng/ml LPS and 250 μg/ml alum, the supernatants were collected and centrifuged at 150*g* for 5 minutes to remove cell debris. The supernatants were diluted with equal volume of buffer A containing 20 mM HEPES-KOH, pH 7.5, 5 mM MgCl_2_, and 0.5 mM EGTA, and were centrifuged at 4600*g* for 10 minutes to precipitate extracellular ASC specks. The extracellular ASC specks were resuspended with buffer A and further purified in a self-generated Percoll gradient at 16,000*g* for 60 minutes at 4°C. After washing extracellular ASC specks with PBS, the number of ASC specks was quantified by counting the GFP fluorescence–positive ASC specks using hemocytometer. For in vivo experiments, mice were administered intranasally 5 × 10^6^ particles of GFP-ASC specks in 50 μl PBS.

### Quantitative real-time PCR.

Total RNA was isolated from WT and *Arf6*^–/–^ airway macrophages by the acid guanidinium phenol chloroform method. cDNA was prepared from 1 μg total RNA by using ReverTraAce (Toyobo) with oligo(dT)_20_ primers. Real-time PCR was carried out using SYBR Green Realtime PCR Master Mix-Plus (Roche) in the Thermal Cycler Dice Real-Time PCR System (TaKaRa). Primer sequences used in this study were as follows: 5′-TCCTAATGAGCGTCCTCCAC-3′ and 5′-TCCTAGGAATGGGTTTTGGA-3′ for *Arf6*; 5′-AACGGCTACCACATCCAAGG-3′ and 5′-GGGAGTGGGTAATTTGCGC-3′ for 18S rRNA.

### Indirect immunofluorescence assays.

Indirect immunofluorescence assays were carried out as previously described (41). Briefly, cells were fixed with 3% PFA for 10 minutes and permeabilized with PBS containing 0.5% Triton X-100 for 3 minutes. After incubating in PBS containing 1% skim milk for 1 hour, the coverslips were incubated with rabbit anti-ASC antibody for 1 hour and then with Alexa Fluor 488–conjugated anti-rabbit IgG antibody (Invitrogen; A11008) and Alexa Fluor 568–conjugated phalloidin (Invitrogen; A-12380) for 30 minutes. To confirm GFP-ASC speck engulfment, cells were fixed with 3% PFA and permeabilized with PBS containing 0.5% Triton X-100, and then the coverslips were incubated with Alexa Fluor 568–conjugated phalloidin for 1 hour. Images were acquired by confocal laser scanning microscopy (LSM700; Carl Zeiss) using × 63 Apochromat objective.

### Statistics.

Statistical significance was tested using either 2-tailed Student’s *t* test or 1-way ANOVA with Tukey’s test. Outliers were excluded by the Smirnov-Grubbs test. *P* values of less than 0.05 were considered significant.

### Study approval.

All in vivo experiments were carried out according to the Guidelines for Proper Conduct of Animal Experiments, Science Council of Japan. The protocols for the mouse experiments were approved by the Animal Care and Use Committee of the University of Tsukuba (19-441).

## Author contributions

SL, AI, TK, YHL, and AK conceived, designed, and performed the experiments. SL and AK analyzed the data. SL, AI, AS, TH, YF, YK, KN, and AK contributed reagents, materials, and/or analysis tools. SL, TK, and AK wrote the manuscript.

## Supplementary Material

Supplemental data

## Version 1 08/23/2021

Electronic publication
