# Supplementary material for: Arf6 exacerbates allergic asthma through cell-to-cell transmission of ASC inflammasomes
Source: JCI Insight. 2021 Aug 23;6(16):e139190. doi: 10.1172/jci.insight.139190 (PMC8410019; doi:10.1172/jci.insight.139190)
Supplement: Supplemental data [file jciinsight-6-139190-s211.pdf]

Supplementary Materials for

Arf6 exacerbates allergic asthma through cell-to-cell transmission of ASC inflammasomes

SangJoon Lee, Akari Ishitsuka, Takahiro Kuroki, Yu-Hsien Lin, Akira Shibuya, Tsunaki Hongu, Yuji Funakoshi, Yasunori Kanaho, Kyosuke Nagata, and Atsushi Kawaguchi*

*Correspondence to: ats-kawaguchi@md.tsukuba.ac.jp

The PDF file includes:

Supplemental Figure 1. Lung tissue sections of HDM-challenged WT and Mϕ-*Arf6* cKO mice.

Supplemental Figure 2. OVA uptake by dendritic cells for antigen presentation in mLN.

Supplemental Figure 3. The amount of IL-13^{+} ILC2 cells in OVA-challenged WT and Mϕ-*Arf6* cKO mice.

Supplemental Figure 4. Isolation of airway macrophages from WT and Mϕ-*Arf6* cKO mice.

Supplemental Figure 5. Purification of extracellular GFP-ASC specks.

Supplemental Figure 6. Extracellular ASC specks-mediated IL-1b production in neutrophils.

Supplemental Figure 7. Effect of SecinH3 on allergic inflammation in Mϕ-*Arf6* cKO mice.

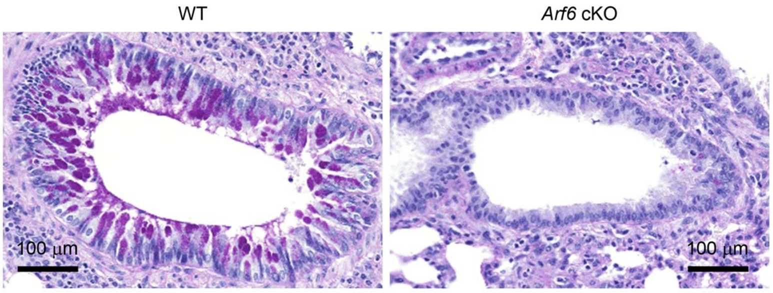

Supplemental Figure 1. Lung tissue sections of HDM-challenged WT and Mϕ-*Arf6* cKO mice. Lung tissue sections were stained with PAS-hematoxylin at day 1 after the last HDM challenge. Data are representative of three independent experiments.

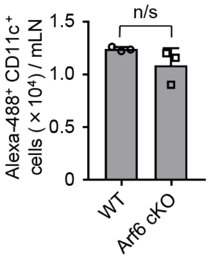

Supplemental Figure 2. OVA uptake by dendritic cells for antigen presentation in mLN. Alexa Fluor 488-conjugated OVA (100 μg) was intranasally administered to OVA-sensitized mice at day 7 after the last immunization. At day 1 after the OVA challenge, the single cell suspensions were obtained from isolated mediastinal lymph nodes (mLN) and were stained with anti-CD45 and anti-CD11c antibodies. The total number of Alexa Fluor 488-positive dendritic cells in mLN was shown (n = 3 mice per group). Each symbol represents one mouse. n/s; not significant.

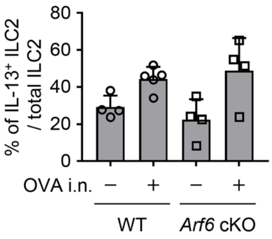

Supplemental Figure 3. The amount of IL-13$^+$ ILC2 cells in OVA-challenged WT and Mϕ-*Arf6* cKO mice.

Cell suspensions were prepared from lungs of WT and Mϕ-*Arf6* cKO mice after OVA challenge. CD45$^+$Lin$^-$CD44$^+$CD90$^+$ST2$^+$CD25$^+$ cells were isolated from the lung cells as ILC2 cells. The isolated ILC2 cells were incubated with Golgi STOP protein transport inhibitor (BD) at 37°C for 4 h and subjected to immunostaining with anti-IL-13 antibody after 4% PFA fixation. The percentages of IL-13$^+$ ILC2 cells to total ILC2 cells were shown. Each symbol represents one mouse (n = 4 to 5 mice per group). The combined results from two independent experiments are shown.

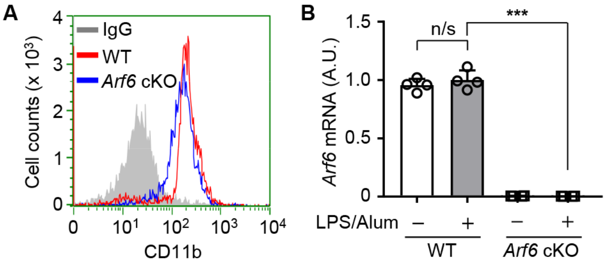

Supplemental Figure 4. Isolation of airway macrophages from WT and Mϕ-*Arf6* cKO mice. **(A)** Airway macrophages in BALF obtained from WT and Mϕ-*Arf6* cKO mice were isolated and the number of CD11b^{+} (Mac-1) cells were counted by flow cytometry. Data are representative of three independent experiments. **(B)** At 36 h post treatment of 200 ng/ml LPS and 250 μg/ml alum, total RNAs were purified from WT and *Arf6*$^{-/-}$ macrophages and subjected to real-time PCR with primers specific for *Arf6* mRNA. Mean ± SD from four independent experiments are shown. n/s; not significant, ****P* < 0.001; two-tailed Student's *t*-test.

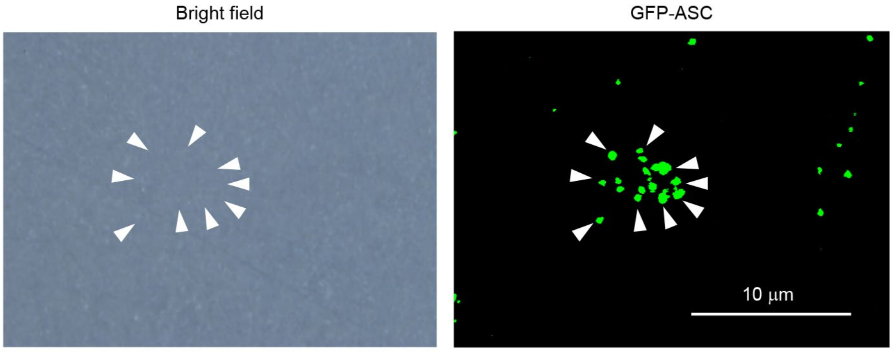

Supplemental Figure 5. Purification of extracellular GFP-ASC specks.

At 48 h post treatment of 200 ng/ml LPS and 250 μg/ml alum, the supernatants of THP-1 macrophages constitutively expressing GFP-ASC was centrifuged to remove cell debris and then subjected to a Percoll gradient to purify extracellular GFP-ASC specks. It was confirmed that there is no contamination of THP-1 cells in the bright field images. Representative images of cell-free purified extracellular GFP-ASC specks are shown.

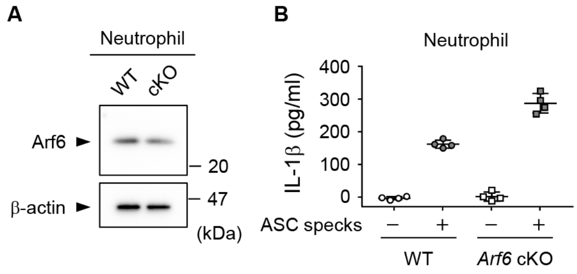

Supplemental Figure 6. Extracellular ASC specks-mediated IL-1β production in neutrophils. Neutrophils were isolated from bone marrow cells by Neutrophil isolation kit (Milteny Biotec; 130-097-658) according to the manufacturer's instruction. **(A)** The expression level of Arf6 in neutrophils isolated from WT and Mϕ-*Arf6* cKO mice was examined. **(B)** IL-1β production in neutrophils isolated from WT and Mϕ-*Arf6* cKO mice was examined by ELISA at 6 h post treatment of 5×10^4 particles of purified extracellular ASC specks.

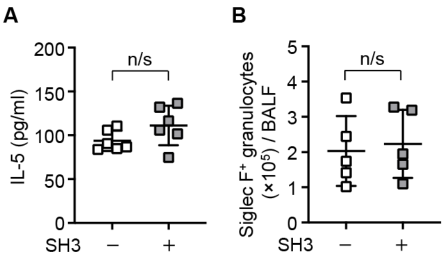

Supplemental Figure 7. Effect of SecinH3 on allergic inflammation in Mϕ-*Arf6* cKO mice.

OVA-immunized Mϕ-*Arf6* cKO mice were intranasally injected with OVA at day 7 after the last immunization. After a 1-day incubation, the mice were intranasally administered with 50 nmol/head SecinH3 (SH3) and then challenged with OVA at days 10 and 13 after the last immunization. The amount of IL-5 (**A**) and the number of Siglec-F^{+} granulocytes (**B**) in BALF were examined at day 1 after the last OVA challenge by ELISA and FACS, respectively (n = 5 to 6 mice per group). Each symbol represents one mouse. n/s; not significant.
